# Affecting behavioural change through empowerment: conceptual insights from theory and agricultural case studies in South Asia

**DOI:** 10.1007/s10113-022-01939-7

**Published:** 2022-06-23

**Authors:** Serena H. Hamilton, Wendy S. Merritt, Lucy Carter, Arnab Chakraborty, Michaela Cosijn, Lilly Lim-Camacho, Rajeshwar Mishra, Geoff Syme, Mahanambrota Das, Dhananjay Ray

**Affiliations:** 1 Institute for Water Futures, Fenner School of Environment & Society, Australian National University, Canberra, ACT Australia; 2 Commonwealth Scientific and Industrial Research Organisation (CSIRO) Land & Water, Canberra, ACT Australia; 3 CSIRO Land & Water, Brisbane, QLD Australia; 4 Professional Assistance for Development Action (PRADAN), Kolkata, India; 5 CSIRO Agriculture and Food, Brisbane, Australia; 6 Centre for Development of Human Initiatives (CDHI), Jalpaiguri, India; 7 College of Sciences and Engineering, University of Tasmania, Sandy Bay, Hobart, Australia; 8 CSIRO Land & Water, Hobart, Australia; 9 Shushilan, Dhaka, Bangladesh

**Keywords:** Self-agency, Human behaviour, Decision-making, Empower

## Abstract

Affecting behavioural change is a common underlying goal across environmental and agricultural sciences, from climate change mitigation and adaptation, biodiversity conservation, water management, to crop diversification. However, many projects fail to drive or sustain change despite sound science and good intentions. This paper draws on existing theories of behavioural change to construct a conceptual framework that explores pathways to initiate and sustain change through the lens of empowerment, self-efficacy and agency. The framework is demonstrated with case studies from a project in India and Bangladesh that examined social inclusion of marginalised and poor farmers in the context of intensifying agriculture. The framework and case studies highlight that a number of conditions are needed to affect meaningful change including that target beneficiaries are suitably motivated, believe in their own capability and power to enact change and have access to the necessary resources. We propose the framework as a tool to help project teams explore the underlying elements of the process of change when designing, implementing and assessing agricultural or environmental projects and interventions. We contend that behavioural and social change needs to be explicitly fostered in such endeavours to achieve better and longer-term outcomes for the people and environment.

**Supplementary Information:**

The online version contains supplementary material available at 10.1007/s10113-022-01939-7.

## Introduction

With many global sustainability issues rooted in human behaviour, one of the key challenges to promoting pathways towards more positive futures is affecting the behaviour and choices of individuals and organisations (Jena and Behera 2017; Steg and Vlek 2009). However, research efforts in environmental and agricultural sciences often overlook the social and psychological factors behind human behavioural change that are related to specific causes of, or potential solutions to, the study problem. This is despite the extensive literature on aspects of behavioural change published over several decades and across varied research domains, including international development, education, health care, psychology and sociology. In order to achieve better outcomes for the environment and society, we assert that researchers working in environmental and agricultural fields need to explicitly address, or at least better understand, behavioural change in the people they target with their research or interventions. Growing appreciation of the need to consider behavioural change is reflected in the body of research using participatory approaches and social learning to promote changes in attitudes and behaviours related to socio-ecological systems (e.g. Étienne 2014; Henly-Shepard et al. 2015; Voinov et al. 2016).

This paper explores behavioural change through the lens of empowerment, self-efficacy and agency, drawing on a set of established theories about behavioural and social change (e.g. Ajzen 1985, 1991; Bandura 1977; Kabeer 1999). The paper is intended to provide insights, for an audience more familiar with biophysical aspects of environmental and agricultural sciences, on how change in individuals and groups can be catalysed and sustained. We introduce a conceptual framework based on the aforementioned social theories to map the process of change at an individual or group level through several pathways, including social learning. It was developed in a project that examined social inclusion of poor and marginalised farmers in agricultural intensification in India and Bangladesh (referred to as the ‘SIAGI’ project). The *empowering change* framework was one of a series of conceptual frameworks developed to produce transdisciplinary understanding of the key factors, issues and interactions of the social and agricultural systems that affected a person’s beneficial inclusion in agriculture. The other frameworks from the project are a *local water management* framework to explore pathways to improve marginalised farmers’ access to and stewardship of freshwater resources (Hamilton et al. 2020) and an *inclusive value chains assessment* (IVCA) framework (Hamilton et al. 2019).

The framework presented in this paper maps out important psychosocial concepts in relation to several types of change in individuals and groups that underpinned local water management and agricultural production and marketing in the SIAGI project, including how the farmers engaged in production (e.g. crop choice, farming practice), in the market (e.g. negotiating prices, approaching new buyers), in their household (e.g. expenditures, diet), within the community (e.g. participation in community-based organisations and activities) or with institutions (e.g. gaining access to entitlements, achieving desired community water quantity and quality outcomes). Although this conceptual framework was developed in the context of addressing equity issues in impoverished communities, we contend that it could be adapted to provide insight on behavioural change across other agricultural and environmental contexts.

## Empowering change framework

The framework presented in this paper draws from behavioural change theories and observed processes in change in the SIAGI project. The development process of this and other conceptual frameworks in the SIAGI project is described in Hamilton et al. (2019). The framework was iteratively developed through project team workshops and discussions, review of project reports and field visits to the communities. The validity and robustness of the framework were tested by applying it to various scenarios across study villages, including different people, at different organisational levels (from individuals, to households and small and larger groups) in varied contexts; this is illustrated in the examples of the framework’s application provided in the “Framework case study application” section (Sect. 3).

### Underlying theory

The framework is centred on empowerment, defined by the World Bank as “the process of enhancing the assets and capabilities of individuals or groups to make purposive choices and to transform those choices into desired actions and outcomes” (Aslop et al. 2006). Power here is considered in terms of the ability to make choices (Kabeer 1999) and exert control and influence over one’s life (Zimmerman 2000). The proposed framework examines empowerment as a dynamic process to enact positive change in various aspects of a person’s life.

The framework emphasises empowerment as a process of change as opposed to a condition or a state. There are two main characterizations of empowerment (Drydyk 2013). In earlier literature, empowerment was considered a relational concept involving the transformation of power relations between individuals and/or groups (Batliwala 1993). The concept has shifted to the more widely accepted notion of being a process whereby a person (or group) increases their capacity to make decisions, generally in the context of being better able to shape their own life and achieve desired actions and outcomes (Aslop et al. 2006; Kabeer 1999). The framework supports both notions by considering the change process as one where an individual or group gains power to exercise choice. VeneKlasen and Miller (2002) note that this can occur through shifts in any of the four forms of power: power within (through sense of self-worth and self-knowledge); power over (e.g. shifts in perceived power imbalances with others); power with (e.g. collective strength through collaboration) and power to (potential to shape one’s life).

The framework comes from the assumption that various levels and types of disempowerment or helplessness are the underlying cause of inertia or a lack of change; this includes lack of motivation, perceptions about lack of control or authority and perceived or real lack of skills, knowledge and resources to enact change. The theory of learned hopefulness suggests that psychological empowerment can be developed through involvement in community organisations and other activities that provide experiences to learn skills and gain a sense of control (Zimmerman 1990).

Human behaviour is highly complex and can be viewed from many levels; therefore, there are numerous existing theories related to behavioural change (Davis et al. 2015). The conceptual framework proposed in this paper does not attempt to explain the intricacies of behavioural change, but rather provides insights on several pathways through which change can be catalysed and sustained. The framework particularly draws on aspects from two well-known theories of behaviour: the theory of planned behaviour (Ajzen 1985, 1991) and self-efficacy theory (or social cognitive theory; Bandura 1977). Ajzen’s theory of planned behaviour postulates that behavioural intention is formed by three critical sets of beliefs held by the individual(s): attitudes towards the behaviour, including the likely consequences; perceptions about social pressure and what others think about the behaviour (normative beliefs) and perceived control over performance of a behaviour. Behavioural intention captures the individual’s motivation or readiness to perform the behaviour. Behaviour results when intention is combined with strong perceived behaviour control (Ajzen 1991).

Self-efficacy, as with perceived behavioural control, is about an individual’s belief in their own capability to perform a behaviour (Ajzen 2002). Perceived self-efficacy is defined as the “beliefs in one’s capabilities to organize and execute the courses of action required to produce given levels of attainments” (Bandura 1998, p.624). This self-efficacy affects a person’s choice of behaviour and the effort they will expend on persisting with the activity if confronted with difficulties (Bandura 1977). Self-efficacy is developed from four main sources: performance accomplishments from personal experiences; vicarious experience from observing others perform the activity; verbal persuasion by others; and physiological and affective states, whereby anxiety, stress or other emotional states affect the judgement of their efficacy (Bandura 1977). With self-efficacy placed at the centre of our framework, we emphasise Bandura’s first three sources of self-efficacy as the main drivers of change in our work, with the assumption that the fourth source, emotional states, is less important for longer-term behavioural change. Whilst the emotional state of an individual and group as well as the community (cultural emotional state) can affect the instigation of change generally, it tends to have less influence over long-term adoption of behaviour which relies on the cognitive realities of doing it. Bandura (1977) notes that the extent of influence of vicarious experience and verbal persuasion on self-efficacy can depend on the characteristics of the person being observed or doing the persuading. In a similar vein, in describing how a person’s desires and standards of behaviour can be conditioned by others, Ray (2006) describes the idea of an ‘aspirations window’, which is populated by ‘similar’ individuals (spatially, economically and socially) who determine a person’s aspirations. The experience of others who lie outside the aspiration window may have little influence on an individual, compared to the experience of those within the window, which is considered more attainable (Ray 2006). This alludes to the importance of social groups such as friends, family and community in influencing behaviour.

Another key concept relevant to empowerment and behavioural change is agency. Agency is “the ability to define one’s goals and act upon them” (Kabeer 1999, p. 438) and is considered the core of the process through which choice is exercised. According to Kabeer (1999), choice also comprises two other key dimensions in addition to agency: resources (including human, social and material) and achievements (i.e. the outcomes of choices). Agency is rooted in one’s belief that they possess the power to make things happen (Bandura 2000), which is critical to exercising control over decisions of change. Self-efficacy is often considered the foundation of agency (Bandura 2000).

### Framework description

**Fig. 1:**
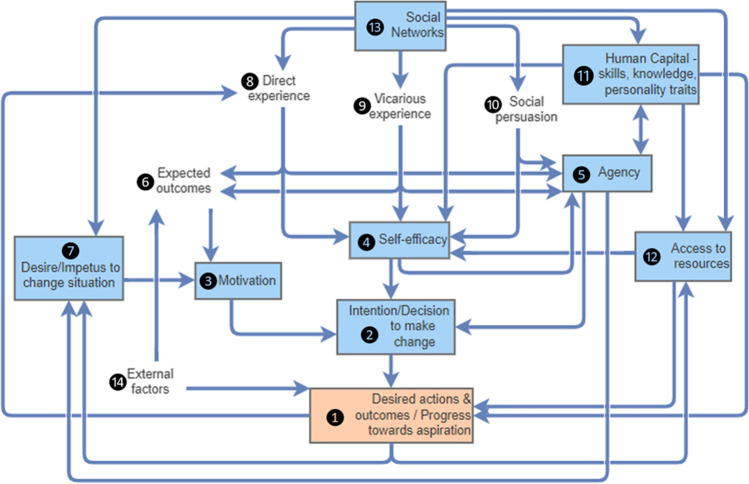
A conceptual framework describing the process of change through empowerment. The diagram shows the numbered concepts (with core concepts in boxes) underlying a person or group’s decision to make change ultimately leading to the final outcome (orange box), and arrows indicate how the concepts are linked. Concepts and key linkages are described in main text (see corresponding circled numbers)

The framework represents the process of change in the form of a flow diagram (Fig. 1), which identifies the key conditions or factors (referred to as concepts) underlying a person or group’s decision to make a change. Circled numbers in the text correspond to the numbered concepts in Fig. 1. The arrows indicate a relationship between concepts which can be positive or negative.

The framework shows several possible pathways that can ultimately lead to the final output of the framework, i.e. the *desired actions and outcomes*/*progress towards aspirations* ❶. The desired outcomes or aspirations of an individual or group can be anything from higher or more consistent income, and money for their children’s education, to non-monetary goals such as improved diet or health, happiness or reduced work hours. In the context of environmental management, examples of desired outcomes may be the recovery of a population of threatened species, and the protection or improved aesthetics of an environmental asset. Below we describe change in general terms of a person performing a specific (new) task, with examples in the agricultural context, but as mentioned above, change can be applied to all possible aspects of a person’s lives and in various contexts.

The *intention/decision to make a change* ❷ is dependent on three main factors: the person’s *motivation* ❸ (‘This is what I want to do’), *self-efficacy* ❹ (‘I am capable of doing this’) and *agency* ❺ (‘I have the power to make this choice’). The strength of these three drivers of change will determine the amount of effort people would be willing to spend in turning this choice into action and persisting in the face of difficulties (Bandura 1977).

*Motivation* ❸ to change is driven by the *expected outcome* ❻ of the change and whether this lines up with the person’s goals and aspirations and their *desire/impetus to change their situation* ❼. Without motivation, there is little incentive to change or to persevere when faced with setbacks. This relationship alludes to the importance of decision makers consulting with potential recipients when designing interventions to ensure investments are in line with the goal of recipients. Furthermore, motivation depends on the centrality or priority of the issue that the change addresses; for example, even if a farmer is interested in practising organic farming, they may not be motivated to shift to this type of farming if their immediate priority is achieving high yields and quicker returns to pay off loans. There may also be consideration of whether there are more effective ways to achieve their goals (e.g. higher income through non-agricultural work).

The *expected outcomes* ❻ of the change can be influenced by what the person has experienced personally (*direct experience* ❽) or has heard or witnessed from others (*vicarious experience* ❾). For example, many farmers in one of the study villages were initially sceptical of the SIAGI project as a consequence of being involved in other projects in the past that were extractive and ineffective. In addition, the expected outcome may also be influenced by factors outside of their control (*external factors*, including social, environmental and other); for example, where outcomes rely on collective effort (e.g. area-wide pest control), expected inaction of other local farmers can demotivate a farmer.

*Self-efficacy* ❹ is the belief in one’s capability to perform a specific task. The framework captures three main sources of self-efficacy (Bandura 1977): direct experience, vicarious experience and social persuasion. *Direct experience* ❽ in undertaking the specific (or similar) task is the most effective way one can develop self-efficacy and assess their capabilities. People can also learn by observing others perform the task (e.g. neighbour growing a new crop) and seeing the outcomes of these tasks (i.e. vicarious experience). *Vicarious experience* ❾ allows the person to compare their own capabilities in relation to others; these social comparisons can help to raise their self-efficacy (‘If they can do it, so can I’). Whilst experiences of success, direct or vicarious, can build efficacy, failure can undermine it. *Social persuasion* ❿ is the encouragement (or discouragement) by others in their social networks, which can help build (or erode) self-efficacy.

The framework also recognises that a person’s *human capital* ⓫ and *access to resources* ⓬ can influence their self-efficacy. Human capital ⓫ includes a person’s skills, knowledge and personal qualities. In addition to skills and knowledge, personal qualities such as assertiveness, problem-solving skills, resilience, creativity, confidence and entrepreneurship can help cultivate self-efficacy, especially in challenging situations. Table 1 lists examples of qualities of individuals and groups and their potential outcomes. *Access to resources* ⓬ captures the resources the individual or their household currently holds as well as those they can gain access to (e.g. a tractor they can lease from a local farmer or business). Relevant resources include the materials, assets, support or services, entitlements and information (including scientific advice) necessary or helpful for performing the given task. The individual’s human capital, as well as their *social networks* ⓭, which includes connections to both formal and informal institutions, can affect their ability to access the resources they need. The social networks create an environment that enables change (or not). We consider an enabling environment as one that consists of informal institutions that are supportive (providing encouragement ❿) and formal institutions that are (i) effective (e.g. provides timely delivery of quality services), (ii) fair in that they are inclusive and equitable and allow participatory or representative decision-making at all levels and (iii) responsive to the needs and requests of marginalised groups (e.g. institutions are willing to communicate with the marginalised people and adapt to their needs and interests). The qualities of institutions are often context-specific as they rely on the people in them.

**Table 1:** Examples of ideal qualities of individuals and groups, and the potential outcomes corresponding to each quality. These qualities were compiled by authors during one of the project workshops

	Qualities	Outcomes
Individual	Assertiveness	Tell providers what I want, and get the support needed
	Problem-solving	Find solutions to overcome issues faced
	Resilience	Cope and recover from difficulties
	Creativity	Find innovative ways to carry out tasks
	Entrepreneurship	Turn ideas into action
	Interpersonal	Able to work well with others
	Readiness	Try new things
Group	Good leadership	Inspire, rally and nurture the team
	Presence of change makers	Create momentum to change
	Inclusive	Everyone can contribute to decision-making
	Shared vision	Efforts focused in the right direction
	Effective teamwork	Work together to define and achieve collective goals and to resolve conflicts as they emerge
	Influential	Attract support, and can affect change
	Reflective	Adaptive learning and more strategic planning
	Linkages to important players	Greater access to facilities and opportunities

The third main driver of change, a sense of *agency* ❺, concerns the belief that one has the power or authority to make the decision. For example, without agency, a female farmer may not expand their production or sell their produce at a marketplace despite being capable and motivated. On the other hand, with agency, a farmer may be willing to speak out to hold local authorities accountable to services they are meant to deliver. Self-perceptions regarding one’s capability to execute the task, i.e. self-efficacy, are paramount to agency (❹ → ❺). There is a two-way relationship between agency and personal attributes such as confidence and assertiveness (❺ ↔ ⓫). Greater agency may also lead to more ambitious goals or aspirations, and thus desire to change their situation (❺ → ❼). A person’s agency can be influenced by their social networks via social persuasion, as well as through previous experience (both direct and vicarious). Social networks ⓭ may impose their shared norms, customs, ideas and rules. Accordingly, social networks can also influence an individual’s aspirations and goals. In addition to personal or self-agency, agency can take on two other forms: collective agency, referring to a group’s collective efforts and power, and proxy agency, where one enlists someone with power and influence to act in their interest (Bandura 2000).

After making the decision, the next step is taking appropriate action. Outcomes rely on applying the right skills and resources (⓫ + ⓬ → ❶), but can also be affected by *external factors* (⓮ → ❶). For example, a new farming practice that is perfectly executed may result in poor yields or low profits if extreme climate events or a market crash takes place. On the other hand, if a new action is successful and helps the individual achieve a given goal or progress towards it, this will feedback into the change process as direct (positive) experience and subsequently strengthen self-efficacy (❶ → ❽ → ❹). Unsuccessful attempts are not necessarily dire, but can undermine self-efficacy especially if it occurs early on in the learning process. Personal qualities such as resilience are particularly important in cases faced with difficulties or risks, as it can lead to persistent effort despite challenges (Wuepper and Lybbert 2017). The outcomes of actions may lead to feedbacks not only to self-efficacy (via direct experience), but also to access to resources (❶ → ⓬) and to the desire to change (❶ → ❼). Farmer collectives in Dhaloguri, one of the SIAGI case study villages in India, for example, were successful in their greenhouse trials of off-season spinach. The spinach sold for high returns, leading to greater financial capacity to expand their greenhouse production. Subsequently, some farmers expressed greater ambition for the village to become the spinach specialist of the region.

The framework can be applied to different stages of the change process, from initiating change to sustaining it. Change is first initiated through the exposure to new ideas (e.g. via vicarious experience), and motivation to change is triggered if expected outcomes match up with the person’s or group’s goals. This motivation needs to coincide with self-efficacy, agency and access to resources for change to occur. This change can be further catalysed by drivers of these factors (e.g. social persuasion, vicarious experience, personal attributes). It is hypothesised that change is sustained if it leads to desired outcomes or progress towards aspirations, thereby reinforcing self-efficacy and agency. Change may be sustained even with unsuccessful attempts if that experience leads to learning that improves self-efficacy.

## Framework case study application

In this section we use three case studies from the SIAGI project to illustrate how the empowering change framework can help explore and articulate behavioural change associated with a project. The SIAGI project covered six villages in the Eastern Gangetic alluvial plains in northern West Bengal, the northern hills of the East India Plateau in southern West Bengal and the coastal zone in south-western Bangladesh. The project focussed on issues faced by the poor and marginalised groups in these villages, in particular small-holder, landless and women farmers and tribal communities, and observed short-term outcomes (1–4 years) of various interventions carried out by the project and two other ‘sister’ projects. Work with the communities was underpinned by an Ethical Community Engagement (ECE) framework, brought into the project by our NGO partners, which emphasised a respectful, empathetic approach to community engagement and treated the development of self-efficacy as the foundation to empower communities to drive their own development agenda (Carter et al. 2021; Mishra et al. 2018).

We examine three case studies of change related to agriculture at a group level:

1. Crop diversification by a women’s farmer collective in Uttar Chakowakheti, West Bengal, India: The farmers in this women’s collective went from growing only one type of crop (rice paddy) once a year to growing a diverse range of crops for income and household consumption. Through growing and selling their vegetables at the market, the women achieved a broad range of outcomes including a greater sense of freedom and happiness, which further encouraged them to mobilise their own resources to expand their agricultural production.
2. Improving freshwater availability through a community-based Water and Silt Management Committee (WSMC) in Khatail, south-western Bangladesh: A WSMC was established to manage the community-owned canals, which had been previously controlled by a few ‘elite’ shrimp farmers. The shrimp farmers used to let saline water enter the canals to create the brackish conditions required for shrimp production, despite most of the community wanting to use the canals to store freshwater to alleviate serious water scarcity issues in the dry season. The community-based WSMC successfully petitioned the local government to stop allowing saline water into the canals, and also built dykes to increase storage capacities in some of the canals. Freshwater is now available for community members in the canals, providing households access to drinking water all-year-round and enabling farmers to grow crops in the dry season.
3. Improving nutrition through women self-help groups (SHGs) in Bankura, West Bengal, India: Women SHGs in two villages in Bankura district, Chakadoba and Hakimsinan, participated in various vision-building and nutrition training programmes to address some of the serious poverty and hunger issues faced in their communities. In addition to enhancing agency and building self-confidence, the women learnt about various aspects of nutrition security and were encouraged to cultivate legumes and vegetables to improve their own and their families’ diets. Through the project NGO, the women and their community were connected with the West Bengal government through the Accelerated Development of Minor Irrigation Project (ADMIP) to develop water assets to support their domestic and irrigation needs. The women’s ability to work with the ADMIP team to determine what irrigation resources they needed and to manage the finances and implementation of the water interventions was crucial in reinforcing agency and self-confidence, as well as building motivation to make change and access the resources needed to act. Household diets have markedly improved across both villages and many farmers have improved their incomes through selling their produce.

More detailed narratives describing the change process in the three case studies can be found in the Supplemental Material.

In all three case studies, behavioural and social change was catalysed through our local NGO partners who worked directly with the communities to provide support and training targeted at their respective issues (i.e. crop diversification, water management and nutrition). As mentioned above, guided by the ECE framework, the overarching aim was to develop self-efficacy (❹ in Fig. 1) of the poor and marginalised farmers in the communities to beneficially engage in agriculture. Self-efficacy was built through training programmes and demonstrations to provide farmers with better knowledge (⓫) and direct experience in the new agricultural practices (❽); organising visits to other farms and villages to expose farmers to new ideas and ways of doing things (❾); and through verbal encouragement, with the NGOs supporting farmers particularly in the early stages (❿). The NGOs also helped to facilitate collective agency and proxy agency (❺), and over time, encouragement and support came from other people with whom the farmers developed relationships (⓭), including government officials, non-SIAGI researchers and external farmers. The three case studies involved community-based groups, thereby representing group-level or collective change; however, if change was considered at an individual level, other members within the respective groups (⓭) were sources of encouragement and learning.

The case studies started with different levels of motivation to change. For example, in the first case study, the women in the Uttar Chakowakheti farmer collective previously had no intention to grow vegetables (no ❼ or ❸), since no other farmers in the village grew crops other than rice. It was not until the demonstration and exposure visit organised by the NGO that they contemplated this change. Whilst the initial impetus (❼) for the women to grow their own vegetables was household consumption, additional income became the primary motivation to grow vegetables after experiencing some success in selling a small amount at the market (additional ❶ and new ❼). In the second case study, the community in Khatail suffered from the lack of freshwater in the dry season prior to the SIAGI project, with households often needing to fetch drinking water from neighbouring villages. The desire to change how water was managed in Khatail was initially high (❼), although the community as individuals had felt powerless to change (low ❹ and ❺). With this high desire to change, the WSMC and community were motivated to try and secure freshwater (❸) despite early difficulties in obtaining support from the local government. The formation of the WSMC, of which 70% of households became members, enhanced unity amongst farmers (⓫) and their strengthened collective agency ultimately enabled them to petition to higher authorities for change (❺).

Outcomes from the changes (❶) in the case studies varied through time and between individuals. In the Uttar Chakowakheti case study, selling vegetables not only provided income, but also gave the women an opportunity to see new things, try new foods and meet and interact with people. Their confidence grew, especially with their produce becoming popular at the markets and other farmers beginning to seek their advice about growing new crops. In the Khatail case study, access to freshwater from the canals enabled farmers to grow dry season crops and meant women no longer needed to travel far distances to fetch water which made them much happier. The additional income enabled households to buy essential items, pay for their children’s education and invest in agricultural intensification with more livestock and crops (further increase in ⓬ and❶). In the Bankura case study, approximately 250 marginal farming households in the two villages started to grow new crops including vegetables, legumes and dry season cereals. Food security—that is the number of months where households are certain they would have at least two meals of food a day— increased across the villages from only 3 to 9 months a year to 9 to 12 months a year, and diets have diversified.

The experiences observed in the case studies were mostly encouraging although the Uttar Chakowakheti case study demonstrated that the change process is not linear and can face setbacks that must be overcome. Despite positive outcomes achieved particularly in the first years, some of the collective’s planned crops failed in early 2020 and left the women feeling somewhat demoralised (❶ not achieved, leading to reduced ❽, ❹ and ❸). Failure resulted from the unexpected rise in market prices of inputs including seeds, which more than doubled in price (⓮), combined with the farmers having insufficient access to financial capital (⓬) due to their limited savings and being denied promised credit from the bank. This highlights how marginalised groups with limited access to resources can be less resilient to adapt when a sequence of adverse events occurs (importance of ⓬, especially in the face of ⓮).

The COVID-19 pandemic has also been a significant external factor affecting the groups (⓮). The pandemic has restricted movement across all regions and led to reduced access to markets and increases in the price of agricultural inputs and transport. Despite the increase in cost of production and other challenges arising from COVID-19 restrictions, farmers across the three case studies continued to report improved agricultural productivity, which helped improve food self-sufficiency for household consumption or maintain some level of income. In Khatail, several farmers reported record profits during this crisis period. With the project interventions occurring before the pandemic, the case study groups had already commenced various types of behavioural and social changes and experienced positive outcomes which helped them build many capacities and resources (e.g. ❹, ❺, ⓫, ⓬ and ⓭) that might have contributed to their resilience to deal with the challenges presented by COVID-19 (⓮).

The case studies also demonstrated how change through empowerment can help foster other types of changes beyond what was initially targeted. Although the NGOs in the project were working with the community-based groups on specific issues, through the ECE process they were helping farmers and community to build their confidence to deal with various challenges. By helping to develop more general aspects of agency and self-efficacy in individuals, rather than, for example, just providing technical solutions to specific problems, the interventions across case studies have led to a broader range of changes and outcomes. In Chakadoba, Bankura, the increased confidence in the women enabled them to seek and successfully access government funding for 30 cattle floors in the village to help reduce risks from dermatological diseases. In Khatail, the WSMC helped increase the voice of community members to speak out and address issues unrelated to water, for example, redressing problems with some livestock-damaging crops.

## Discussion

Through developing this framework, it was our intention to explore and communicate the processes of change as observed in our study villages, in particular to understand what factors helped to catalyse and sustain change. The iterative development of the framework provided a boundary object that helped our NGO partners articulate their knowledge and practice and contribute to the team’s learning process. This validated our NGO partners’ practice and also helped them to better understand the behavioural change process, including why past interventions were not successful, and appreciate their role in the change process (see Merritt et al. 2022). The framework represents a model of change from a research and practitioner perspective. Our approach to conceptual modelling can potentially be adapted to explore the farmer’s perspective in line with participatory or companion modelling (Ducrot et al. 2015; Gourmelon et al. 2013), noting that such a study is likely to generate a more context and problem-specific model. Our intention was to develop a more general model of the change process. However, we acknowledge that the change pathways represented in our framework may be biassed towards those observed in our project, which may have been influenced by the socio-cultural context of the study villages as well as our research activities. To overcome this inherent bias, we grounded the framework in established literature and, when reviewing the framework, encouraged the team to think about their observations from other projects (in other regions as well as other sectors) where intended change in communities may or may not have been realised. The framework was also developed for local-scale problems that could be addressed with local solution. In its current form, the framework captures a set of combined hypotheses about behavioural and social change that were derived from select theories (e.g. Ajzen 1985, 1991; Bandura 1977; Kabeer 1999) and remains open to further testing.

Many projects and interventions across environmental and agricultural sciences fail to drive or sustain change and achieve the desired impact (Kamoto et al. 2013). To achieve positive outcomes, projects need to explicitly foster behavioural change and get buy-in from the people impacted by and who can impact the effectiveness of the interventions. In the case of the SIAGI project, we needed buy-in from the local farmers who were both the beneficiaries and the primary agents of change. In the case of environmental problems, such as river water quality, the beneficiaries (e.g. downstream water users) are not necessarily those from whom we want behavioural change (e.g. upstream water users or land holders). Interventions need to consider the individuals or groups involved, and specifically their aspirations or desires, their capabilities including access to resources and, importantly, their beliefs about their own abilities. In some cases, the desired change may require change in other players (e.g. extension workers, local government officials or policy-makers), in which case the framework can be applied again with the new actor and their motivations, efficacies and constraints as the focus. The same principles would apply; however, the work needed to create buy-in, for example, may need to happen on a larger scale at an organisational or institutional level.

Through improved understanding of the process of behavioural change, we argue that projects and interventions can be better designed if they target the concepts that are most underdeveloped and also consider the pathway(s) of impact. For example, hands-on learning not only increases human capital by increasing knowledge but also provides experiential learning that contributes to self-efficacy and agency. Designing interventions with the change process, as described in the framework, in mind can help ensure they are more effective at realising and maintaining positive outcomes for communities and their environment. The framework can also help to strengthen individual and social learning when used in conjunction with monitoring and learning processes, or structured learning processes such as described in Ensor and de Bruin (2022).

This paper has focused on behavioural change pathways to empower marginalised groups or individuals to enact positive change for themselves. Across the case studies described in Sect. 3, we observed shifts in all four forms of power identified by VeneKlasen and Miller (2002)—power within, power over, power with and power to. Firstly, the Uttar Chakowakheti and Bankura case studies demonstrated change by instilling power and thus confidence *within* the women to produce and market crops. The second type of power shift was seen in the Khatail case study in relation to control *over* the canal waters. There, change occurred through redressing the power imbalance held by the local elite (i.e. shrimp farmers) who sought to ‘capture’ community resources for their own benefit at the expense of the majority. This was driven by the community members uniting to achieve collective strength *with* one another, the third type of power (akin to collective agency). In each of the case studies, the marginalised groups increasingly gained the fourth type of power, i.e. confidence and power *to* make choices and exert changes in their own lives, as desired outcomes were achieved through the learning process. Whilst the opportunity for the marginalised farmers to diversify their crops, or unite to seek support from government authorities, was ostensibly available to them prior to the project, the lack of self-efficacy and agency within these groups presented major barriers to change without the intervention of the SIAGI project and its sister projects.

The example of ‘elite capture’ by the shrimp farmers in Khatail demonstrates that not all forms of empowerment are positive. Such abuse of power by certain individuals or groups has been widely recognised as a key threat to community development initiatives, which can lead to resources being misappropriated and diverted away from intended beneficiaries (Lund and Saito-Jensen 2013; Platteau 2004). It is critical that the broader socio-political and cultural contexts are carefully considered when designing projects and interventions (van Kerkhoff and Pilbeam 2017). In our framework, this consideration falls under ‘social networks’, which describes both the formal and informal institutions connected to the individuals or groups. Social networks can be seen as a major driver of change, linking to several of the other factors in the framework, including desire to change, direct and vicarious experience, social persuasion, human capital and access to resources, as well as helping to enable proxy agency. Whilst on one hand, this positions social networks as an important enabler of change—indeed the SIAGI interventions can all be viewed as changes that occurred through social networks in the form of the community-based groups (i.e. farmer collectives, WSMC and SHGs) and their connections to, for example, the local NGO, external farmers, government officials and researchers. On the other hand, some social networks can be impediments of change, who work against the best interests of the individuals or groups. It is therefore important that problems are examined from a broader perspective that captures the social environment and considers whether appropriate enabling conditions are in place before solutions are proposed or implemented, and that proponents or funders of interventions play an active role in monitoring and mitigating potential threats to project outcomes, such as elite capture (Platteau 2004) or competition for resources by other parties.

It is evident that the creation of social change derives from altered relationships within a social system operating at differing levels, from individuals through families to community-based organisations and government. We selected social theory applicable to potential community change from discussions and community agreement (through ethical community engagement), and they were used to inform the change model presented in this paper. The advantage of applying this selective theory is that others can use the insights created by the literature surrounding each component, to provide insights into their own community practice or problem. The approach is a development of Varela’s concept of social technology (Varela 1971, 1977) whereby selected established theory is used pragmatically to underpin targeted societal change. This approach is now about 50 years old and relied at the time on a medical model of the professional intervening to a dysfunctional group or organisation. This attracted some justifiable criticism in relation to the ethics pertaining to informed consent and the limitations of the experimental basis of the theory used by Varela (e.g. Agyris 1975). However, the evidential basis of social theory has improved significantly over 50 years and the introduction of collaborative problem definition and resolution with the community as shown in this project provides an ethical basis on which social technology can be developed (Singer and Glass 2015).

## Conclusion

This paper has set out to encourage non-social scientists to think about behaviour and social change in real life settings. Whilst we acknowledge a much larger body of work that can be further explored (e.g. Davis et al. 2015), the proposed conceptual framework captures established social theory around behaviour and change that can be used as a tool to help think through and discuss pathways to change when designing, implementing and assessing interventions or projects. When designing an agricultural development or environmental intervention, the framework suggests the need to first ensure the core elements of change are in place (i.e. motivation, self-efficacy, agency and access to the necessary resources) in order to instigate and sustain the desired change. To use the framework to strategically intervene in the change process, researchers or practitioners should engage with the target groups to identify which concepts represented in the framework are underdeveloped or absent. Such an exercise will help to determine what activities or resources are required to promote and support the desired change. The most effective intervention will depend on the individual case and its context. The case studies presented highlighted that developing more general aspects of self-efficacy and agency in the target groups and helping build their social networks were not only critical for achieving the intended outcomes, but also helped foster other positive changes in their lives. When developing interventions, thinking beyond the specific problem at hand and developing more general capacities may help lead to a broader range of outcomes.

Affecting and sustaining behavioural change require corresponding shifts in how research-for-development (R4D) programmes are funded and evaluated (Leeds and Palaia 2021). For donors, this may include providing sufficient flexibility for research partnerships to remain agile to changing workplans in response to new learning and shifts in research or community’s focus. For participating research organisations, enabling researchers to move from theoretical and research-driven approaches to a more transdisciplinary approach to science, where the production of knowledge and learning is shared more equally amongst all actors, continues to be a challenge to traditional ways of working (Fritz et al. 2019; Rau et al. 2018). This may require the creation of alternative incentive mechanisms to support researchers in pursuing transdisciplinary research collaborations and producing a broader range of science outputs. More focus on the people involved and affected by interventions and their behaviour in both research and practice is critical for ensuring that intended changes are initiated and sustained so that better outcomes for the environment and society are achieved.

## Supplementary Information

Below is the link to the electronic supplementary material.

Supplementary file1 (DOCX 31 KB)
