# Supplementary material for: Affecting behavioural change through empowerment: conceptual insights from theory and agricultural case studies in South Asia
Source: Reg Environ Change. 2022 Jun 23;22(3):85. doi: 10.1007/s10113-022-01939-7 (PMC9218702; doi:10.1007/s10113-022-01939-7)
Supplement: Supplementary file 1 — Supplementary file1 (DOCX 31 KB) [file 10113_2022_1939_MOESM1_ESM.docx]

# Supplemental Material - Case study narratives

### **Women’s farmer collective in Uttar Chakowakheti, West Bengal, India**

Uttar Chakowakheti is a tribal village in West Bengal, with predominantly small and marginal farming households. A women’s farmer collective of 12 members was formed in 2016 with support from the project’s local NGO partner, CDHI. Through CDHI, the farmers received training on diversifying their crops and polyhouse (low-cost version of greenhouse) cultivation [providing ❽ and ⓫], with field demonstrations as well as exposure visits to farmers in neighbouring villages [❾]. Prior to the formation of the collective, the farmers only grew one type of crop – monsoon paddy (rice) – once a year. Now these farmers grow a diverse range of crops all-year-round including cabbage, broccoli, beans, lady finger, tomato, potato, mustard and capsicum [❶].

Prior to the project, these female farmers had no intention to grow vegetables [no ❼ or ❸] – in fact they reported that no one in the village grew vegetables. Their change started with the village demonstrations and exposure visits to other farms [❽ and ❾], and encouragement from CDHI [❿]. From there, they first tried to grow vegetables primarily for household consumption [initial ❼] but also successfully sold a small amount at market [additional outcome ❶]. Over time, they continued selling at the market [reinforced ❽→❻+❹+❺] and their produce became renowned for being fresh and chemical-free, and often sold out at the market early in the day.

Before growing and selling their own vegetables, many of these women farmers were dependent on their husbands to go to the market to buy produce. Since, they have enjoyed going to the market every week– it has given them a greater sense of freedom and happiness. It is not only a source of income, but gives them an opportunity to see new things, try new foods, meet and interact with people and get ideas [broad range of positive outcomes ❶]. Other farmers now seek their advice about growing new crops. Although their first polyhouse was damaged by a cyclone, the women’s collective mobilized their own funds to build another polyhouse [greater ❹, ❺ and ⓬]. Subsequent to the training of the women’s collective and other groups, farmers throughout the village are now growing all sorts of vegetables. For one of the farmers in the women’s collective, the profits from farming had become sufficient enough that her husband no longer needs to migrate seasonally for work [❶].

Despite positive outcomes achieved in the first few years of the project, in early 2020, the farmers in the women’s collective reported setbacks that demoralized them somewhat [❶ not achieved leading to poor ❽, ❹ and ❸]. The vegetables in the polyhouses were growing well, however some of their planned in-field vegetable crops had failed. Some of the production inputs had suddenly gone up in price, including seeds, more than doubling [⓮]. They were also unable to access promised credit from the bank to purchase the seeds [⓬]. This meant they were unable to sow as much land as planned, and the few crops they were growing in the field suffered from nutrient deficiencies due to insufficient fertilizer applications [poor outcome ❶]. The failure of their field crops was due to the unexpected rise in market prices of inputs [external factor ⓮] and the farmers in the collective having insufficient financial capital [limited access to resources ⓬]. This more recent incident highlights the potential vulnerability of marginalised groups who have limited access to resources. Insufficient financial capital can easily lead to individuals or groups being less resilient and able to adapt when a sequence of adverse events occurs [importance of ⓬]. However, later in 2020, the women farmers achieved good returns on capsicum and coriander produced in their polyhouse and open fields [❶].

In early 2021, their field crops, particularly maize, successfully germinated and had good initial growth, but were subsequently lost from livestock grazing [mixed outcomes ❶]. The women in the farmer collective have become increasingly technically sound, and continue to learn about the whole ecosystem of farming [⓫ improving]. The collective is gradually improving their agricultural resilience by applying their learnings. As a result of the COVID-19 pandemic, the collective faced difficulties in collecting seeds and other inputs due to markets closing down and restricted transport facilities [⓮]. The women farmers managed to access the inputs they required using personal relationships with local retail sellers [⓭→⓬], but this came at a 20-30% increase in cost of production. Despite the increased input costs from the pandemic, overall productivity increased in 2020/21 due to an increase in cultivation area [❶]. They gained some profit from paddy, mustard, tomato, brinjal, and other leafy vegetables 2020, but overall profit margins were less compared to 2016 to 2019. In the past year the women have also been involved in a collective enterprise that produces and sells bio-inputs for farming; this small entrepreneurship development in this village and others was facilitated by the sister project (DSI4MTF) and supported by the international organisation 3ie through the University of Birmingham.

### **Water and Silt Management Committee in Khatail, south-western Bangladesh**

The village of Khatail is located along the coastal delta of south-western Bangladesh. Prior to the SIAGI project, the village faced serious issues around freshwater availability for both domestic and livelihood needs that the majority of the community wanted addressed [high ❼]. With freshwater the main constraint to agricultural intensification, our local NGO partner, Shushilan, facilitated the formation of a Water and Silt Management Committee (WSMC) to manage canals that surround the village. Despite being community-owned, these canals were previously controlled by a few shrimp farmers with strong links to government officials, who would allow saline water to enter the canals to produce the brackish conditions required for shrimp farming. The vast majority of the community (>95%) were in favour of freshwater in the canals, but felt powerless to change how they were managed. Shushilan supported the community in developing a constitution for the WSMC, which outlined rules and regulations aimed at ensuring inclusivity, transparency and accountability. More than 70% of households in the village became members of the WSMC. Through engagement processes, the NGO also encouraged and helped the marginalized community members develop self-efficacy and agency [❿ →❹+❺]; see Hamilton et al. (2020) for details around the local water management in Khatail.

The WSMC and community initially had difficulties in obtaining the support of the local government to stop the ingress of saline water into the canals in the dry season. Furthermore, tensions between shrimp farmers and crop farmers seemed to intensify following the WSMC formation. However, unity amongst most farmers in the community improved [⓫ and ⓭ improved] with the interventions, including the engagement processes, and their strengthened collective agency enabled them to speak up to higher authorities [❺]. The WSMC successfully coordinated a mass petition to the local government to stop allowing saline water into the canals, gaining support from leaders and community members from Khatail and neighbouring villages. They achieved non-objection to store freshwater in the canals [❶]. The WSMC also mobilised resources to build dykes to increase storage capacities in some of the canals. During the last dry season (2020/21), there was a shortage of canal water due to the reduced depth of the canal from siltation and an increased number of farmers, many of whom were growing water-intensive rice crops in the dry season. In response to this water shortage, the WSMC re-excavated the canal and also encouraged farmers to grow less water-intensive crops, providing members with pumpkin and watermelon seedlings.

In addition, the WSMC managed to garner support from government officials to resolve conflicts over open livestock rearing. Traditionally, livestock were allowed to roam freely, and would enter planted fields and damage crops, particularly in the dry season; this practice is no longer allowed. WSMC members also successfully negotiated with banking institutions to help farmers access agricultural loans; most loans taken out have already been paid off [examples of other outcomes ❶ from improved ❹+❺].

Access to freshwater from the canals has enabled farmers to grow crops in the dry season, and improved access to water for drinking, domestic use and livestock [❶]. Women are happier that they no longer need to travel long distances to neighbouring villages to fetch water in the dry season. Many farmers have had successful dry season crops, notably watermelon, and the additional income has enabled many households to save money, pay off loans, pay for their children’s education, make improvements to their home, as well as invest in agricultural intensification with more land (purchased and leased), livestock and crops [further increase in ⓬ and❶]. Increased production in the dry season has also increased the amount of agricultural wage labour work available to villagers. This has provided access to work for many local women (ratio of women to men labourers is 3:2), enabling them to contribute income to their household [❶].

The recent COVID-19 pandemic has presented farmers in Khatail with a few challenges related to the restricted movement across the region, which has led to: some difficulties in accessing seeds, fertilizer and pesticide to purchase; selling agriculture products; and increased transportation costs, which have almost doubled [⓮]. Some of the temporary restrictions on movement were imposed by the local administration to reduce the risk of spread of COVID-19, and coincided with the watermelon harvest (between February and April 2021). The WSMC members advocated to the local administration and local officials for more flexibility in the restrictions to allow farmers to sell their products to distant markets. The local administration subsequently allowed agricultural traders and growers with trucks to transport watermelon to other markets, including in Dhaka, [challenges overcome via ⓭], bringing returns on investments of 500 to 600%. Although, livelihood opportunities, income and food security across Bangladesh has decreased during the COVID-19 pandemic, the farmers in Khatail as a whole seemed to have bucked the trend, achieving good harvest and earnings during the crisis period [❶ despite ⓮].

## **Women Self Help Groups, Bankura, West Bengal, India**

This case study involves women self-help groups (SHGs) in two villages in the Bankura district of West Bengal - Chakadoba and Hakimsinan. During the early visits to the villages by the project team in 2017, the effect of extreme poverty and hunger was highly visible to researchers. In both villages, almost all women and children (especially girls) were malnourished, with women suffering from anaemia and other nutrient deficiencies. Together with our local NGO partner, PRADAN, the SIAGI project team jointly developed a nutrition sensitive agriculture (NSA) strategy, devised to improve the food environment in the villages, guided by Ethical Community Engagement (ECE). The primary objective was to devise an evidence-based NSA program that over time could be scaled to other districts.

Through PRADAN, the project worked towards enabling women to take control of their futures to achieve enduring outcomes, instilling a greater sense of confidence and self-esteem in the women and facilitating a rediscovery of hope in their ability to change their condition [developed ❼ and built ❸+❹+❺]. The SHG women members were engaged in vision-building trainings to create an inclusive society, to visualise a desired future state for their society where SHGs proactively looked beyond the self and reached out to others (especially those vulnerable and marginalised) thereby influencing the development discourse in the community [⓭→❿+⓫]. The steps of the broader engagement process included: enhancing individual members’ own sense of agency and self-esteem [❹+❺]; sensitizing women to the need to come together and create robust social capital [⓭]; building a clear and shared articulation of the institutional goals; gap identification and planning [⓫]; collaboration with other stakeholders (e.g. Water Users Associations, agricultural scientists and West Bengal ADMIP) [⓭]; and co-learning [⓫]. PRADAN focussed on the three key indicators of nutrition security: a) *Food access* through improved access to nutritious foods on-farm, increased availability and lower prices of diverse nutritious foods in markets, improved food safety, and income; b) *Care practices* through empowerment of women and through incorporating behaviour change communication; and c) *Health environments* through management practices that protect natural resources (water in particular), and safeguard against health risks.

Through various training programs, women learnt about the importance of good health and nutrition, particularly through a balanced diet [increase ⓫]. In many households, meals had previously consisted of mostly rice. The women also participated in intensive field training in growing “nutrition gardens” (home gardens with fruits and vegetables of high nutritive value) [❽ and ⓫] to help diversify their household diets as well as to have produce to sell in order to buy supplementary food items. They were also provided with some agricultural inputs such as seeds [⓬]. In addition, the women were encouraged to cultivate legumes in the formerly fallowed uplands so that there were sufficient pulses for their household throughout the year [❿→❺→❼]. The women also received training in the concept and process of marketing, teaching them about what to cultivate, when and how [⓫]. They were taken to different markets with their products where they spoke with the businessmen, understood the workings of the market and the factors that determine the price of the products that they send from the villages. This helped them to strategize the timing of sending the products to the market and the packaging process [⓫].

The project also involved creation of physical assets through collaboration with government departments in the villages to increase access and availability of water for irrigation [⓭→⓬]. Initially PRADAN acted as a bridge connecting the women to the relevant government officials. Once the women started gaining confidence, they were encouraged to establish linkages with the government on their own [⓭]. As a result of this increased confidence, the communities have improved access to government initiatives and resources [⓬]. For example, the community in Chakadoba were able to access government funding for 30 cattle floors in the village, which help the women to maintain cleanliness, protecting them from dermatological diseases [❶].

A broad range of positive changes [❶] has been reported in the two villages since the community engagement and training programs have taken place. Firstly, many women are now growing vegetables and pulses (250 households have reported growing new crops), and their household diets are subsequently more diversified. Food security (number of months where they are certain they would have at least two meals of food a day) of households across the villages has increased from 3 to 9 months a year to 9 to 12 months a year. Many farmers have increased their income through selling their produce. And many women, for the first time, have been able to buy items (e.g. dresses, ornaments) exclusively for themselves. The majority of women who participated in the training programs have reported that they are now comfortable interacting with outsiders, including market players. The nutrition gardens adopted by many households has reduced their dependency on the market for buy vegetables for consumption, which has been particularly helpful during the COVID-19 crisis [❶ despite ⓮].
